# Human blood metabolites and gastric cancer: a Mendelian randomization analysis

**DOI:** 10.1186/s12876-024-03576-2

**Published:** 2024-12-30

**Authors:** Chao Zhang, Dao Lai Huang, Kun Zhou, Jin Tao Cai, Dang Liu, Ming Hao Tan, Guan Yu Zhu, Xiang Hua Wu

**Affiliations:** 1 Guangxi Medical University, Nanning, 530021 Guangxi China; 2 Guangxi Key Laboratory of Enhanced Recovery After Surgery for Gastrointestinal Cancer, Nanning, 530021 Guangxi China; 3 Department of Gastrointestinal Gland Surgery, The First Affiliated Hospital of Guangxi Medical University, Qingxiu District Nanning, 22 Shuangyong Road, Guangxi, 530021 China

**Keywords:** Blood metabolites, Causality, Gastric cancer, Mendelian randomization

## Abstract

**Background:**

Gastric cancer (GC) remains one of the predominant malignant tumors within the digestive tract, yet its underlying biological mechanisms remain elusive. The primary objective of this study is to delineate the causal relationship between circulating metabolites and GC.

**Method:**

The primary Mendelian randomization (MR) analysis was based on three large GWAS datasets. While the inverse variance weighted served as the primary analysis technique for investigating causal relationships, additional sensitivity analyses were facilitated through methods such as MR-PRESSO, the weighted median, and MR-Egger. Subsequently, replication, meta-analysis, and multivariable MR were executed using another GC GWAS.

**Results:**

The results of this study indicated significant associations between three metabolites 3-methyl-2-oxovalerate (OR 5.8, 95%CI: 1.53–22.05, *p* = 0.0099), piperine (OR 2.05, 95%CI: 1.13–3.7, *p* = 0.0175), Phe-Phe dipeptide (OR 0.16, 95%CI: 0.03–0.93, *p* = 0.0409) and GC.

**Conclusion:**

The present study provides evidence supporting a causal relationship between these three circulating metabolites and GC risk. Elevated levels of 3-methyl-2-oxovalerate and piperine may increase the risk of GC, while Phe-Phe dipeptide may have a protective effect. By integrating genomics and metabolomics, we offer a novel perspective on the biological mechanisms underlying GC. Such insights have the potential to enhance strategies for the screening, prevention, and treatment of GC.

**Supplementary Information:**

The online version contains supplementary material available at 10.1186/s12876-024-03576-2.

## Introduction

Gastric cancer (GC) is a leading malignancy of the digestive system, ranking fifth in global cancer incidence and fourth in cancer-related mortality. In 2020, over 1 million new GC cases were reported, resulting in approximately 768,793 deaths worldwide [1]. Improving the prevention and early identification of GC is a vital strategic focus.

Metabolic reprogramming is increasingly recognized as a hallmark of cancer, enabling tumor cells to meet heightened demands for energy, macromolecular synthesis, and survival signals [2]. In GC, various metabolic pathways are implicated in tumor initiation and progression. For instance, alterations in amino acid metabolism—particularly glutamine utilization—supply building blocks for nucleotides and lipids while sustaining the tricarboxylic acid cycle to support rapid cell proliferation [3]. Xenobiotic metabolism, including the overexpression of cytochrome P450 enzymes, contributes to the production of reactive oxygen species (ROS), lipid peroxidation, and DNA damage, thus amplifying inflammation and carcinogenesis [4, 5]. Dysregulated lipid metabolism, exemplified by bile acid imbalances, disrupts gastric mucosal integrity and activates pro-inflammatory signaling (e.g., IL-6/JAK1/STAT3), creating an environment conducive to malignant transformation [6, 7]. Concurrently, aberrant carbohydrate metabolism—manifested by heightened glycolysis and pentose phosphate pathway activity—enhances lactate production and ribose sugar availability, fueling rapid growth and immune evasion [8–10]. These interrelated metabolic alterations not only shape the tumor microenvironment but also provide potential targets for early interventions and novel therapies.

Within this context, metabolomics—the comprehensive assessment of metabolites in biological specimens—offers a powerful approach to identify biochemical signatures associated with GC. Observational studies have begun to link specific metabolites to GC risk. For example, decreased tryptophan and increased phenylacetylglutamine levels were identified in GC patients [11], supporting earlier findings [12]. Another study reported significant reductions in cholesterol and certain fatty acids in GC patients, suggesting that GC cells consume fatty acids for membrane synthesis and energy [13]. However, these observational findings alone cannot disentangle whether such metabolic changes are causal determinants of GC or merely byproducts of the disease process. Traditional observational studies are often limited by confounding factors, reverse causation, and a lack of insight into underlying mechanisms.

Advancements in high-throughput sequencing have enabled genome-wide association studies (GWAS) to uncover genetic determinants involved in disease pathways. Certain single nucleotide polymorphisms (SNPs) significantly influence serum metabolite levels. For example, SNP rs1260326 in the *GCKR* gene has been associated with alterations in triglyceride and glucose levels, impacting components of metabolic syndrome and type 2 diabetes risk [14]. SNP rs174547 in the *FADS1* gene affects polyunsaturated fatty acid levels, influencing inflammation and potentially cancer risk [15]. Additionally, SNP rs1440581 in the *PPM1K* gene has been linked to branched-chain amino acid levels, which may play a role in cancer development due to their involvement in cell growth and proliferation [16]. These genetic associations provide a foundation for exploring the causal relationships between metabolites and diseases.

Mendelian randomization (MR) is a statistical approach that uses genetic variations to explore whether a factor, such as a blood metabolite, directly influences the risk of disease [17, 18]. It is often compared to randomized controlled trials (RCTs) because genetic variations are randomly passed from parents to offspring, minimizing the influence of other factors and helping establish the direction of the relationship. Prior research has executed GWAS on metabolites, equipping subsequent investigations to discern causal relationships between metabolites and diseases, particularly by harnessing metabolite-centric GWAS data in MR studies [19].

Several studies in MR research have explored the association between exposure factors such as sleep patterns [20], smoking [21, 22], alcohol consumption [21, 22], obesity [23], and blood lipid levels [24], with GC. However, research focusing on the relationship between blood metabolites and GC remains sparse. The causal link between blood metabolites and GC has not been definitively established. Therefore, this study leverages the availability of GWAS data on 486 human blood metabolites to systematically explore their causal relationships with GC using MR approaches.

## Materials and methods

### Study design

**Fig. 1:**
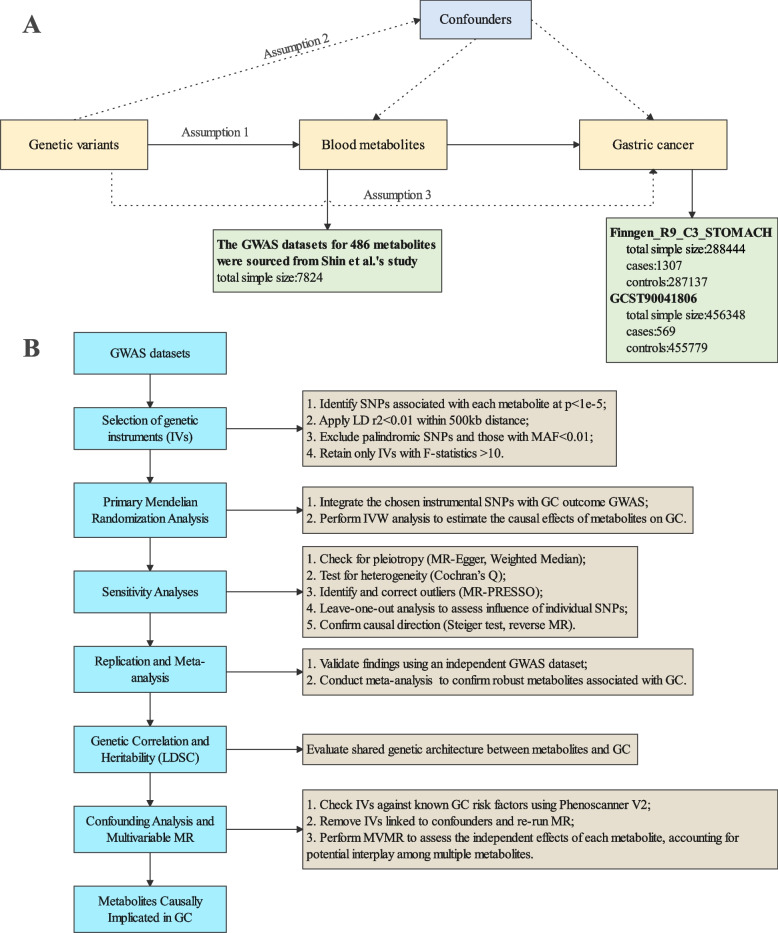
Conceptual framework and methodological workflow of the Mendelian randomization study. SNPs: Single nucleotide polymorphisms; LD: linkage disequilibrium; MAF: minor allele frequency; IVW: Inverse Variance Weighted; LDSC: Linkage Disequilibrium Score; MR: mendelian randomization. **A** presents conceptual model illustrating the three core assumptions in MR: Assumption 1: the genetic instruments must have a strong association with the exposure; Assumption 2: the genetic instruments should not be associated with the outcome or any confounders; Assumption 3: the genetic instruments should affect the outcome solely via the examined exposure. Panel **B** displays analytical pipeline for the MR study. Instrumental variables are selected from metabolite GWAS data using strict criteria. These IVs are then integrated with GC GWAS data for the primary MR analysis to estimate causal effects. Sensitivity analyses (MR-Egger, Weighted Median, Weighted/Simple Mode, Cochran’s Q, MR-PRESSO, leave-one-out) assess pleiotropy, heterogeneity, outliers, and single-SNP influence, as well as confirm causal direction (Steiger test, reverse MR). Replication and meta-analysis validate the findings in independent data, LDSC checks genetic correlation, and confounding analysis plus MVMR refine the results. The final step identifies metabolites with robust causal evidence for involvement in GC

In this research, we meticulously evaluated the causal association between human circulatory metabolites and GC risk via a two-sample MR design (Fig. 1). MR studies rely on three key assumptions to ensure valid causal inference: (1) Relevance: The genetic variants used as instrumental variables (IVs) must be strongly associated with the exposure of interest. (2) Independence from Confounders: These genetic variants should not be related to other factors, such as lifestyle or environmental exposures, that could independently affect both the metabolite levels and gastric cancer risk. For example, a variant linked to both metabolism and obesity could introduce bias. (3) Exclusivity: The genetic variants must affect gastric cancer risk solely through their impact on the metabolite levels and not via other pathways. Sensitivity analyses, such as MR-Egger regression, are often applied to check for violations of these assumptions and ensure the robustness of causal estimates. Genetic data for human blood metabolites and GC were sourced from distinct GWAS datasets to avoid sample redundancy. The study design and analysis methods were performed in line with the STROBE-MR guidelines for MR studies.

### GWAS data for human blood metabolites

The GWAS datasets for 486 metabolites were sourced from Shin et al.'s study [19]. These complete summary statistics are accessible on the Metabolomics GWAS server (http://metabolomics.helmholtz-muenchen.de/gwas/). The GWAS analysis encompassed 7,824 individuals from the TwinsUK and KORA cohorts, covering approximately 2.1 million SNPs. Of these 486 metabolites, 177 remain unidentified with unspecified chemical properties. The remaining 309 recognized metabolites are categorized into eight metabolic groups, as delineated by the Kyoto Encyclopedia of Genes and Genome database: amino acids, carbohydrates, cofactors and vitamins, energy, lipids, nucleotides, peptides, and xenobiotic metabolism.

### GWAS data for GC

The preliminary analysis utilized GWAS data from the FinnGen Consortium's C3_STOMACH dataset [25], encompassing 1,307 GC cases and 287,137 healthy controls, predominantly from the Finnish population. This dataset can be accessed at https://www.finngen.fi/en. For replication and meta-analysis validation of the initial findings, the study employed the Inverse Variance Weighted (IVW) analysis on a cohort (accession number: GCST90041806) sourced from the GWAS Catalog. This cohort includes 569 GC cases and 455,779 healthy controls from the European population and is available at https://www.ebi.ac.uk/gwas/ [26].

### Instruments selection

In this research, we implemented a systematic approach to select appropriate genetic variants as proxies for metabolites. Initially, we identified SNPs related to serum metabolites with a significance threshold set at *p* < 1e-5, ensuring both rigor and a robust number of SNPs for subsequent analysis. We then isolated independent genetic variants by establishing a linkage disequilibrium threshold at r^2^ < 0.01 within a 500 kb radius, referencing the European 1000 Genomes Project. Palindromic SNPs with intermediate allele frequencies (e.g., A/T or G/C alleles) were excluded to prevent analytical ambiguity, as these SNPs have complementary alleles that are indistinguishable on the forward and reverse strands. Additionally, SNPs with a minor allele frequency below 0.01 were omitted due to concerns about their statistical reliability.

The chosen genetic variants were then assessed for their efficacy as proxies for the 486 metabolites based on the explained variance (R^2^) and F-statistic. IVs with F-statistics under 10 were deemed weak and were thus removed. The refined set of SNPs was extracted from the outcome GWAS data, excluding SNPs associated with the outcome (*p* < 1e-5). Any missing SNPs in the outcome GWAS dataset were disregarded. We ensured the final set comprised independent and credible genetic variants, adhering to the previously detailed criteria. The MR analysis was then executed on metabolites with at least three retained SNPs.

### Primary analysis

We evaluated the causal link between 486 blood metabolites and GC using the IVW approach. Frequently employed in MR research, this method combines the Wald ratios from each SNP to produce a unified estimate [27]. IVW operates under the assumption of no horizontal pleiotropy among SNPs [28]. Given this, IVW offers the most precise estimation of causal effects, thus serving as the principal analytical tool in this study for initial causal effect evaluation.

### Sensitivity analysis

Pleiotropy occurs when a single genetic variant influences the outcome through multiple biological pathways rather than solely through the exposure, potentially biasing the IVW estimates. Heterogeneity arises when different genetic instruments produce inconsistent effect estimates, reducing confidence in the overall MR results. To address these issues, we performed a series of sensitivity analyses on metabolites showing significant initial findings (IVW p < 0.05). The analyses involved (1) the use of Weighted Median (WM), MR-Egger, Weighted Mode, and Simple Mode to reevaluate the causal link between metabolites and GC and to determine pleiotropy in genetic variants based on their intercept. Specifically, MR-Egger regression adjusts for pleiotropy, even when all instruments are invalid [29], while WM operates under the premise that over half the instruments are valid [30]. The primary results are derived predominantly from IVW, WM, and MR-Egger methods. Weighted Mode and Simple Mode are used exclusively to assess the causal estimates' direction and magnitude. (2) Cochran's Q statistic evaluated the heterogeneity of the selected genetic variants. A result of *p* < 0.05 or I^2^ > 25% in Cochran's Q test indicates heterogeneity among IVs [31]. (3) The MR-Pleiotropy RESidual Sum and Outlier (MR-PRESSO) method identified and adjusted for outliers and horizontal pleiotropy. When discernible outliers were detected, IVW, MR-Egger, and WM analyses were repeated post-removal. (4) A leave-one-out analysis gauged the influence of individual SNPs on overall results. (5) Additionally, to confirm the correct causal direction, we conducted reverse MR and the Steiger test. The Steiger test compares the variance in the exposure and the outcome explained by the selected genetic instruments. If the IVs explain more variance in the outcome than in the exposure, this suggests potential reverse causation. By contrast, if the IVs explain more variance in the exposure, it supports the hypothesized causal direction (exposure to outcome). Reverse MR then re-examines the association by swapping the roles of exposure and outcome. If reversing the direction does not produce a similar causal signal, it further substantiates that the original direction is correct [32, 33]. The inferred causal direction may be compromised if the explained variance of IVs in GC surpasses that of blood metabolites. All MR analyses employed the TwoSampleMR packages (version 0.5.7) and MRPRESSO packages in R software (version 4.3.0), with *p* < 0.05 denoting statistical significance.

To further assess the statistical power of our MR analysis, we utilized an online calculator (https://sb452.shinyapps.io/power/) which determines power grounded in asymptotic theory [34]. With a Type I error rate set at 0.05, power was calculated using the R^2^ of IVs, the proportion of GC cases, and the odds ratio (OR) from IVW analysis.

### Replication and meta-analysis

To validate the robustness of the metabolites identified in our research process, we incorporated an additional, independent GWAS dataset in the IVW analysis. A subsequent meta-analysis was conducted to identify the definitive candidate metabolites associated with GC causality. Due to the use of only two GWAS datasets for the meta-analysis, which makes it challenging to assess heterogeneity, we employed a random effects model to minimize analytical errors. The meta-analysis was performed using the meta packages in R software (version 4.3.0).

### Evaluation of genetic correlation and heritability

If there is any genetic correlation between the exposure and the outcome, it can potentially bias the MR analysis results. While SNPs associated with GC were excluded in the prior MR analysis, SNPs not directly related to the outcome can also play a role in GC development. The Linkage Disequilibrium Score (LDSC) regression measures shared heritability by performing chi-squared tests on SNPs linked with both the exposure and the outcome [35]. To uphold the foundational assumption of MR studies, we employed LDSC to evaluate the genetic correlation between GC and metabolites.

### Confounding analysis and multivariable MR analysis

We conducted sensitivity analyses to comprehensively assess pleiotropy in the MR results and detect any violations of MR assumptions. Nonetheless, a few residual confounding SNPs might persist. We scrutinized the IVs of chosen metabolites using the Phenoscanner V2 website (http://www.phenoscanner.medschl.cam.ac.uk/) [36], examining if any SNP was linked to established GC risk factors, such as helicobacter pylori [37], smoking [38], alcohol intake [39], BMI [40], and diabetes [41]. SNPs significantly associated with these confounders (*p* < 1e-5) were excluded, and a subsequent MR analysis was conducted to ascertain the robustness of the results.

The effect of individual exposure on the outcome was evaluated using two-sample MR studies, excluding the influences of alternate exposures. In contrast, multivariable Mendelian randomization (MVMR) analyzed the impact of each exposure while adjusting for others [42]. In our research, we utilized MVMR to account for interplays among the detected metabolites and ascertain their isolated causal effects on GC. MVMR analyses were performed using MVMR, TwoSampleMR, MendelianRandomization, and MRPRESSO packages in the R software (version 4.3.0).

## Results

### Strength of the IVs

Based on the IVs selection method described above, we incorporated 485 metabolites into the MR analysis, excluding glutamate with fewer than 3 SNP. The IDs and reference files for 486 metabolites are detailed in Table A1. The count of SNPs for the selected metabolites varied between 3 and 478. Notably, all SNPs had F-statistics>10, ensuring no weak IVs in the selected set. The harmonized data is provided in Table A2.

**Table 1:** Sensitivity analysis for the causal association between blood metabolites and GC

		**WM**		**MR-Egger**		**Heterogeneity**		**Pleiotropy**		**Steiger test**		
**Metabolites**	**N**	**OR (95CI%)**	***p-value***	**OR (95CI%)**	***p-value***	**Q(I**^**2**^)	***p-value***	**Intercept**	***p-value***	**Causal_direction**	***p-value***	**Power**
**Lipid**												
linoleate (18:2n6)	17	0.11 (0.01–0.94)	0.0435	0.59 (0.00–430.16)	0.878	3.76(0%)	0.999	−0.026	0.6	TRUE	3.1E-80	1
hexadecanedioate	25	1.17 (0.60–2.28)	0.644	1.14 (0.56–2.32)	0.727	15.58(0%)	0.903	0.022	0.182	TRUE	7E-214	1
taurochenodeoxycholate	13	1.68 (0.88–3.18)	0.115	1.40 (0.62–3.15)	0.433	5.3(0%)	0.947	0.014	0.568	TRUE	7.7E-98	1
**Peptide**												
gamma-glutamylglutamate	10	0.49 (0.21–1.14)	0.0962	0.39 (0.08–1.98)	0.291	3.57(0%)	0.937	0.012	0.793	TRUE	3.4E-69	1
phenylalanylphenylalanine	4	0.20 (0.02–1.55)	0.122	0.23 (0.00–51.42)	0.649	0.96(0%)	0.812	−0.01	0.895	TRUE	9.1E-25	1
**Amino acid**												
3-methyl-2-oxovalerate	33	8.93 (1.20–66.37)	0.0323	9.29 (0.65–133.83)	0.112	34.08(6.09%)	0.368	−0.007	0.69	TRUE	2E-211	1
**Xenobiotics**												
piperine	11	1.66 (0.73–3.76)	0.223	1.26 (0.33–4.84)	0.746	10.59(5.60%)	0.39	0.028	0.448	TRUE	6.4E-59	1

Table 1 displays a sensitivity analysis to validate the association between specific blood metabolites and gastric cancer risk. It details the odds ratios with 95% confidence intervals for each metabolite, calculated using various Mendelian Randomization methods: Weighted Median, MR-Egger, and the Steiger test, which confirms the direction of causality. It also evaluates heterogeneity and pleiotropy, which assess the variability of genetic instruments and whether SNPs influence outcomes via pathways other than the metabolite in question, respectively. The number of SNPs used as instrumental variables for each metabolite is provided. *P*-values are given for each analysis, indicating the statistical significance of the findings

**Table 2:** Provides genetic correlation and heritability estimates for metabolites concerning their relationship with gastric cancer

	**Genetic Correlation**			**SNP-based Heritability**		
**Metabolite**	**Rg**	**Se**	***p*-value**	**h**^**2**^	**se**	***p*-value**
3-methyl-2-oxovalerate	0.021	0.598	0.9714	0.317	0.062	2.75E-07
piperine	−0.433	1.097	0.6931	0.096	0.064	0.1345
phenylalanylphenylalanine	4.134	7.141	0.5626	0.003	0.102	0.9782

### Primary and sensitivity analysis

**Fig. 2:**
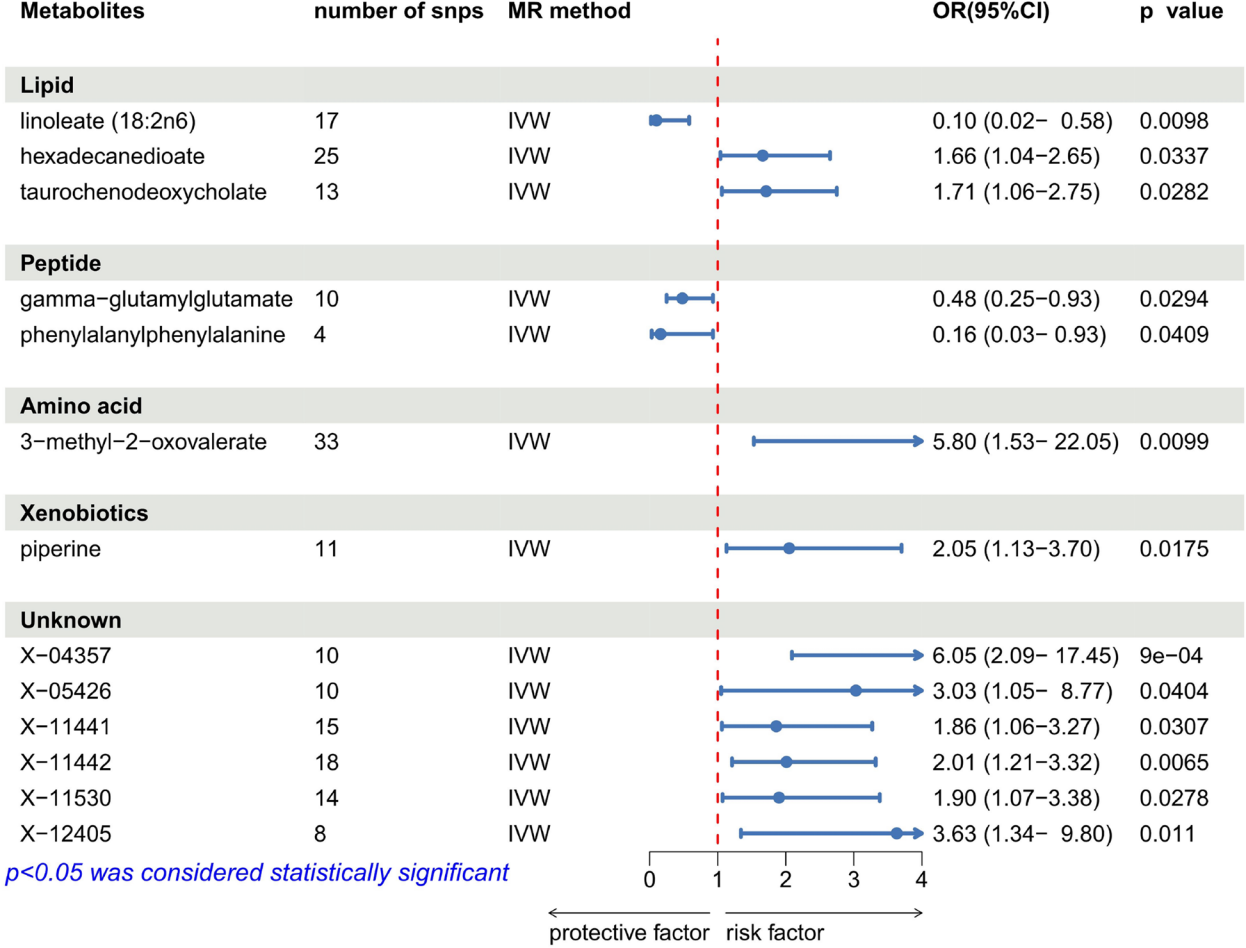
Forest plot for the causal relationship between metabolites and gastric cancer derived from IVW. MR, mendelian randomization; OR, odds ratio; CI, confidence interval; IVW: inverse variance weighted. Forest plot illustrating the causal relationship between various blood metabolites and gastric cancer, as derived from the IVW Mendelian Randomization analysis. Each metabolite is grouped by category (Lipid, Peptide, Amino acid, Xenobiotics, and Unknown) and represented with its respective number of instrumental SNPs, the MR method used, and the OR with 95% CI. The red dashed line at OR = 1 indicates the null effect, with points to the left suggesting a protective effect and points to the right indicating an increased risk. Metabolites with statistically significant associations (*p* < 0.05) are highlighted, including protective factors like phenylalanylphenylalanine (Phe-Phe dipeptide) and risk factors such as 3-methyl-2-oxovalerate and piperine

**Fig. 3:**
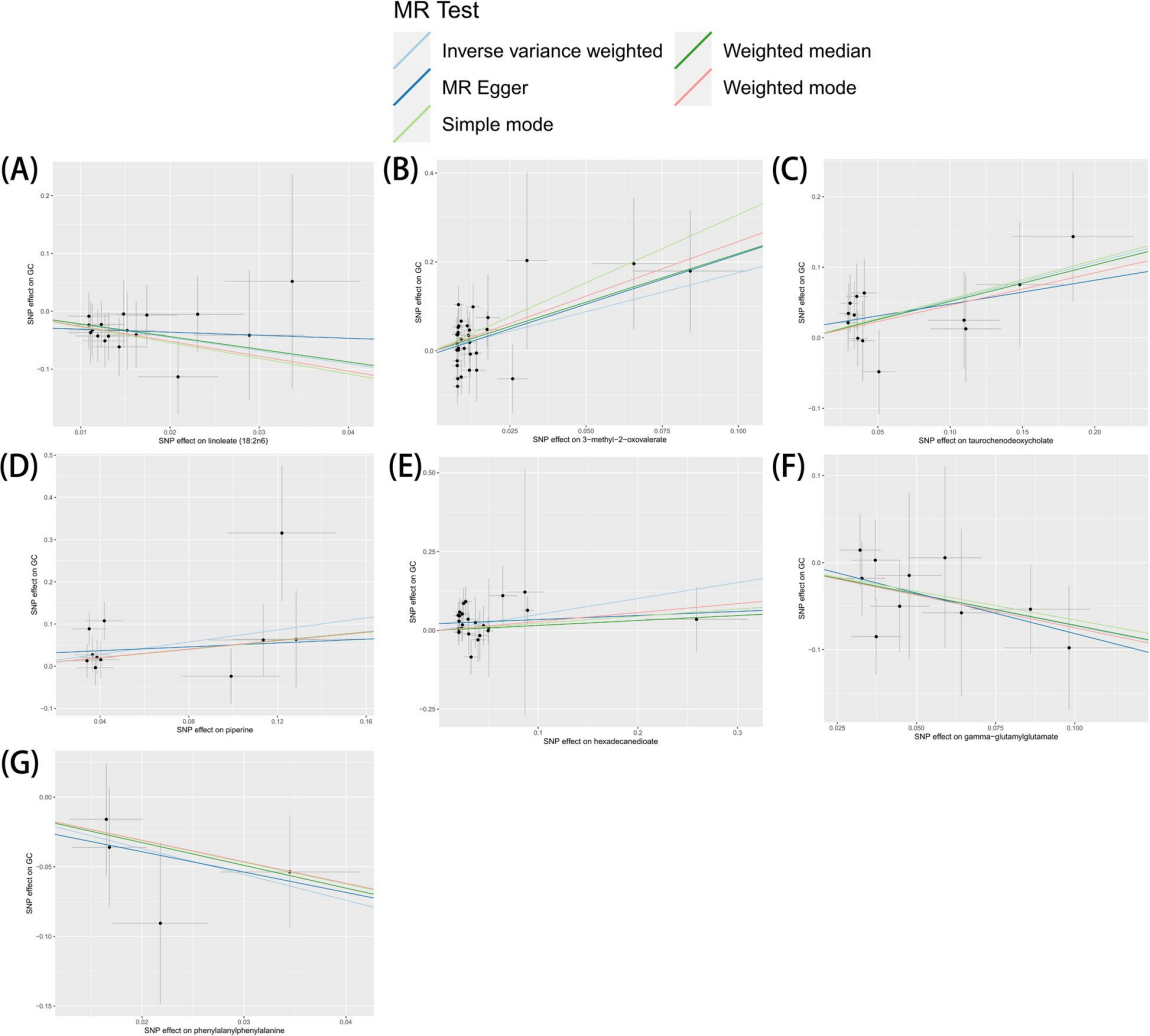
Scatterplot for the significant association (*P* < 0.05) between metabolites and gastric cancer. MR: mendelian randomization; SNP, single nucleotide polymorphism. Each panel (**A**-**G**) represents a scatterplot showing the association between SNPs and gastric cancer risk for a specific metabolite, as analyzed using multiple MR methods. The x-axis indicates the SNP effect on each metabolite, while the y-axis shows the SNP effect on GC risk. Different MR methods are color-coded for comparison: Blue line: Inverse Variance Weighted; Green line: MR Egger; Gray line: Simple mode; Red line: Weighted mode; Pink line: Weighted median. These plots highlight the consistency of causal estimates across various MR methods, with a line slope indicating the direction and magnitude of the association between each metabolite and gastric cancer risk

Through the IVW method, 13 metabolites were identified as having potential causal effects on GC (Fig. 2). Out of these, 6 metabolites have unknown chemical identities. In subsequent analyses, we will focus solely on the analysis of these 7 known metabolites. The remaining 7 identified metabolites were classified like lipid, amino acid, peptide, and xenobiotic based on their chemical nature. Subsequent sensitivity analyses retained all 7 metabolites. The IVW-derived estimates yielded a *p*-value < 0.05. Additionally, the estimates obtained through IVW, WM, MR-Egger, Weighted Mode, and Simple Mode methods were consistent in direction and magnitude, as depicted in Fig. 3. No outlier SNPs were identified by MR-PRESSO (Table A3), and Cochran's Q test indicated no heterogeneity among the selected SNPs, with the MR-Egger intercept test showing no evidence of pleiotropy, confirming the robustness of our analyses (Table 1). Additionally, LOO analysis suggested that individual SNPs did not bias the MR estimates (Figure A1). All MR analyses demonstrated a statistical power greater than 0.8, further validating the reliability and stability of our findings. The results of reverse MR analysis and Steiger tests on metabolites with statistical significance obtained from the primary analysis suggested that the causal relationships of the mentioned metabolites with GC were unaffected by reverse causality (Table A4). Consequently, these 7 metabolites were shortlisted for subsequent examination.

### Replication and meta-analysis

**Fig. 4:**
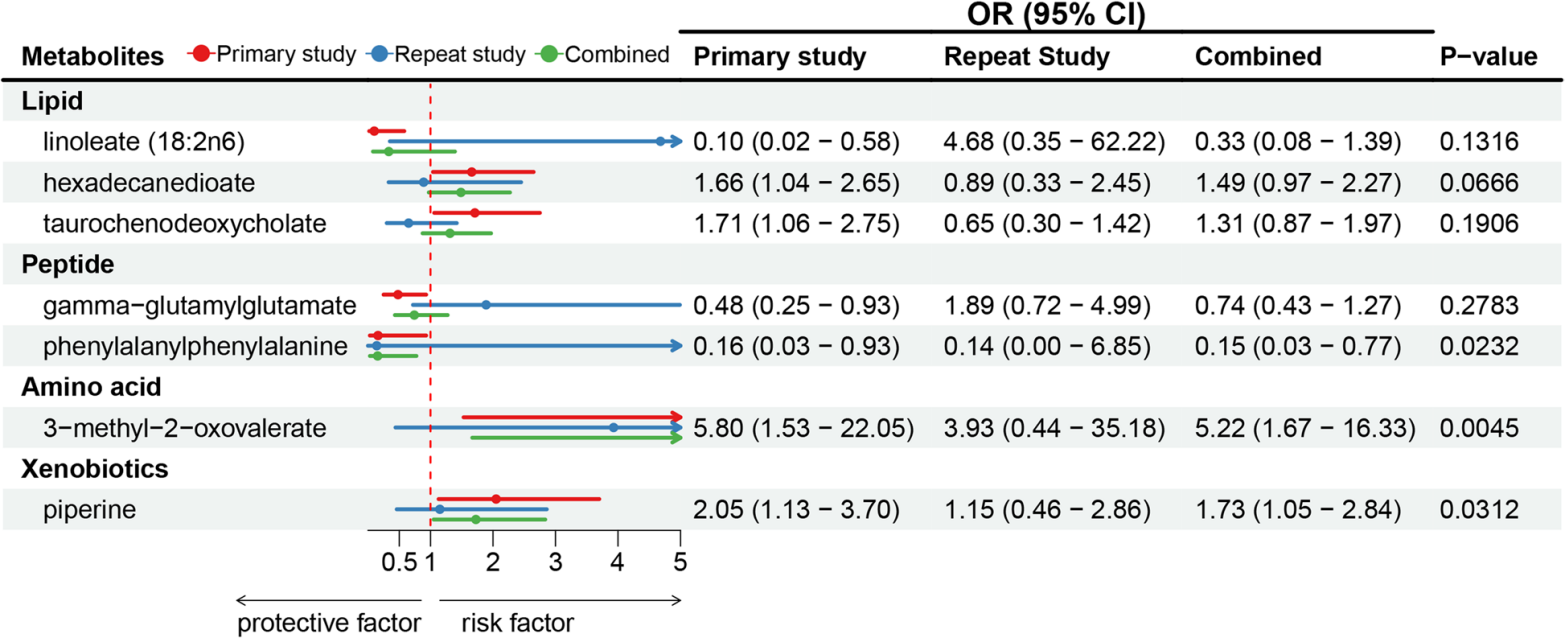
Meta-analysis of significantly associated (IVW derived *p* < 0.05) between metabolites and gastric cancer. OR, odds ratio; CI, confidence interval. This forest plot presents the OR with 95% CI for the association between specific metabolites and gastric cancer, as determined by Inverse Variance Weighted Mendelian Randomization analysis. Each metabolite is shown with results from the primary study (red), repeat study (blue), and combined meta-analysis (green). The red dashed line at OR = 1 represents the null effect, with values to the left suggesting a protective effect and values to the right indicating an increased risk. Metabolites with statistically significant combined results (*p* < 0.05) include phenylalanylphenylalanine (Phe-Phe dipeptide), 3-methyl-2-oxovalerate, and piperine, which are identified as potential factors influencing gastric cancer risk

To enhance the reliability of our findings, we reanalyzed using an alternative GC GWAS dataset. Although these results lacked statistical significance due to variations in sample size and population demographics, three metabolites—3-methyl-2-oxovalerate, piperine, and phenylalanylphenylalanine (Phe-Phe dipeptide)—exhibited consistent effect directions in both the initial discovery and replication analyses (Table A5). Moreover, the meta-analysis affirmed the reliability of causal estimates for these three metabolites in association with GC (Fig. 4).

### Evaluation of genetic correlation and heritability

LDSC-based estimates showed minimal genetic correlation between GC and 3-methyl-2-oxovalerate, piperine, and phenylalanylphenylalanin, indicating that MR assessments are not influenced by common genetic elements. Subsequently, we employed LDSC to compute the SNP-based heritability for three metabolites. The SNP-based heritability results suggested significant genetic variability in the levels of 3-methyl-2-oxovalerate, indicating that genetic factors partially influence variations in this metabolite's levels across the population (Table 2).

### Confounding analysis and MVMR

In our study, while the sensitivity analyses have rigorously excluded SNPs potentially skewing the estimates, we further scrutinized the SNPs associated with the seven metabolites to determine any correlation with prevalent GC risk factors, such as helicobacter pylori, smoking, alcohol consumption, BMI, and diabetes. This assessment was in line with the basic assumption 2, emphasizing that IVs should not be related to confounders. From our investigation, the metabolites 3-methyl-2-oxovalerate and piperine collectively encompassed 4 SNPs linked to these common risk factors (Table A6). As anticipated, after removing these SNPs and re-conducting the MR analysis, the estimated values remained statistically significant: 3-methyl-2-oxovalerate (OR 6.62, 95%CI: 1.72–25.45, *p* = 0.0059), piperine (OR 2.18, 95%CI: 1.17–4.10, *p* = 0.0148).

**Fig. 5:**
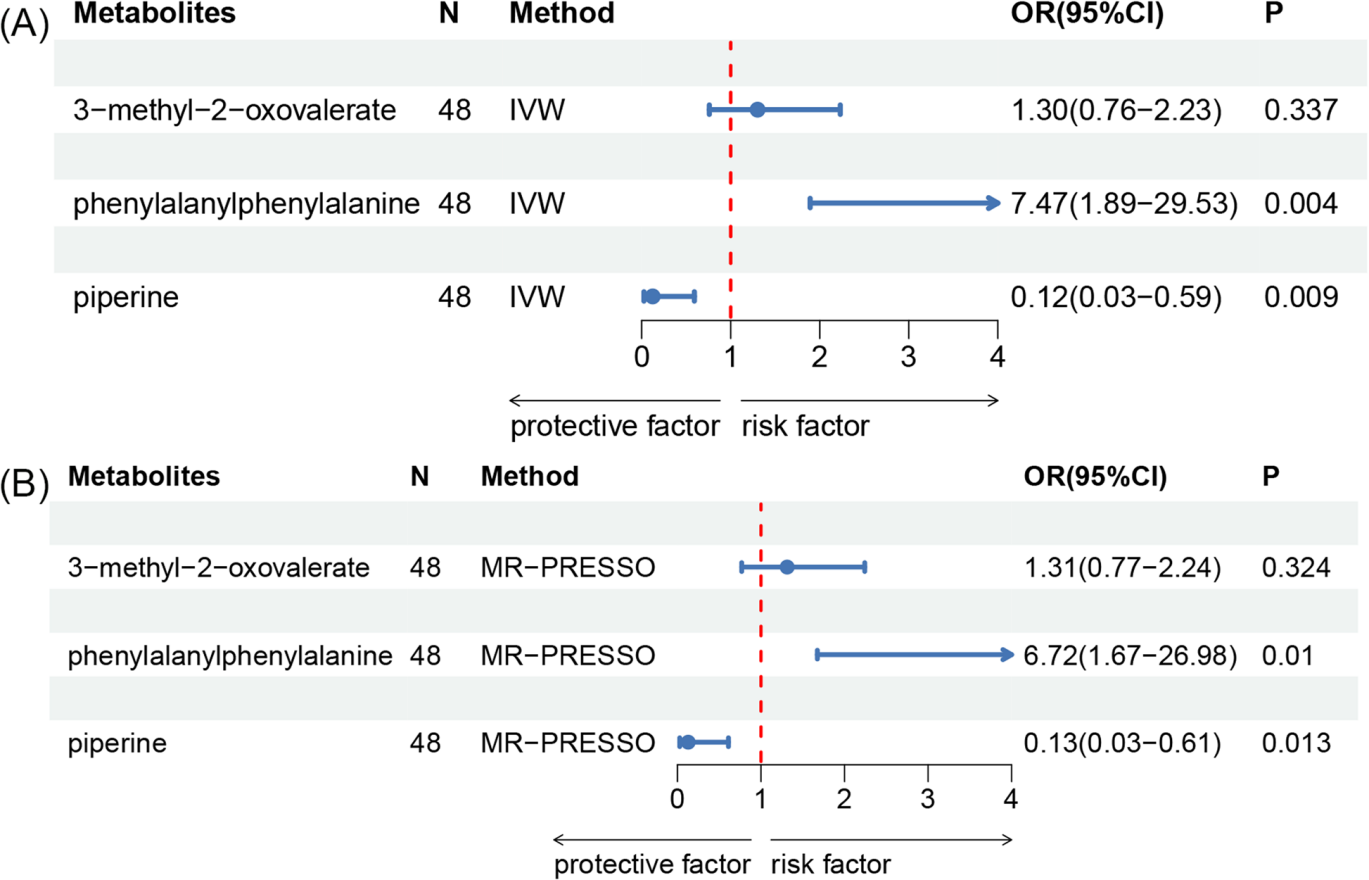
Multivariable Mendelian randomization analysis of the final identified blood metabolites. OR, odds ratio; CI, confidence interval; IVW, inverse variance weighted; MR-PRESSO, MR-Pleiotropy RESidual Sum and Outlier. **A** presents the IVW method results, while **B** displays results from the MR-PRESSO method. After accounting for interactions among the seven metabolites, both MVMR estimates indicate that phenylalanylphenylalanine (Phe-Phe dipeptide) and piperine levels have a direct influence on gastric cancer risk, with statistically significant associations (*p* < 0.05) observed in both methods. OR with 95% confidence intervals are provided for each metabolite, with the red dashed line at OR = 1 representing the null effect. Values to the left of this line suggest a protective effect, while values to the right indicate increased risk

After accounting for the interactions among the seven metabolites, both the MVMR estimates using IVW and MR-PRESSO methods suggest that the levels of Phe-Phe dipeptide and piperine have a direct influence on GC (Fig. 5).

## Discussion

In the present study, we analyzed two extensive GWAS datasets and utilized a stringent MR approach to investigate the association of blood metabolites with GC. Our findings suggest that a genetic predisposition to elevated levels of 3-methyl-2-oxovalerate and piperine heightens the risk of GC. Conversely, elevated genetically determined levels of Phe-Phe dipeptide correlate with a reduced risk of GC. MVMR analyses indicate that Phe-Phe dipeptide and piperine exert a direct influence on GC. To our knowledge, this is the first MR study utilizing comprehensive blood metabolite GWAS data to investigate its potential causal link with GC. This is the first MR study to systematically explore the causal relationship between blood metabolites and GC using large-scale GWAS data. By minimizing biases such as confounding and reverse causation, our findings provide novel insights into the metabolic mechanisms of GC and identify potential biomarkers and therapeutic targets.

Metabolic dysregulation is increasingly recognized as a central driver in GC pathogenesis, shaping tumor growth, survival, and immune evasion through complex alterations in energy and nutrient utilization [43]. By leveraging naturally occurring genetic variations as instrumental variables, MR enables more reliable causal inference than traditional observational studies, effectively minimizing confounding and reverse causation. This methodological strength provides clearer etiological insights into the metabolic pathways underlying GC risk and progression.

Our findings reveal that elevated levels of 3-methyl-2-oxovalerate correlate with a heightened risk of GC, and our results indicate substantial genetic variability in the levels of 3-methyl-2-oxovalerate. To date, only a handful of studies have delved into the role of circulating 3-methyl-2-oxovalerate in tumor initiation and advancement. Notably, the metabolism of valine, leucine, and isoleucine has been frequently linked to cancer progression [44, 45]. 3-methyl-2-oxovalerate, a derivative of the branched-chain amino acid metabolism (specifically valine, leucine, and isoleucine biosynthesis), stands as a branched-chain keto acid (BCKA) offshoot of isoleucine.

Branched-chain amino acids (BCAAs) and their corresponding keto acids play significant roles in cellular metabolism and signaling pathways. Elevated levels of BCAAs and BCKAs have been associated with enhanced activation of the mammalian target of rapamycin signaling pathway, promoting cell growth and proliferation [46, 47]. In cancer cells, increased BCAA catabolism can support tumor growth by providing energy and biosynthetic precursors [8]. Specifically, 3-methyl-2-oxovalerate may influence the metabolic reprogramming of cancer cells, favoring glycolysis over oxidative phosphorylation, known as the Warburg effect [48, 49]. This metabolic shift facilitates rapid ATP production and generates lactate, which acidifies the tumor microenvironment, promotes angiogenesis, and suppresses anti-tumor immune responses [8]. Clinically, elevated levels of 3-methyl-2-oxovalerate may hold potential as a metabolic biomarker for early detection of GC or as a potential therapeutic target to inhibit cancer metabolism. Moreover, elevated levels of 3-methyl-2-oxovalerate may be associated with increased insulin resistance [46], a condition linked with type 2 diabetes and an elevated risk of GC [49, 50]. Insulin resistance contributes to hyperinsulinemia, which enhances tumorigenesis by activating insulin-like growth factor 1 (IGF-1) signaling, a pathway that promotes cell proliferation and inhibits apoptosis. Additionally, insulin resistance induces chronic low-grade inflammation, characterized by elevated cytokines such as TNF-α and IL-6, further accelerating GC progression [50, 51]. Elevated levels of 3-methyl-2-oxovalerate may serve as a potential metabolic biomarker for early detection of GC, enabling improved risk stratification and facilitating timely interventions. Its association with metabolic reprogramming and systemic metabolic disorders suggests a role in shaping the tumor microenvironment, making it a promising target for therapeutic strategies. Targeting pathways linked to 3-methyl-2-oxovalerate, such as branched-chain amino acid metabolism and insulin resistance-related signaling, could offer novel approaches to inhibit tumor growth and progression. However, the complexity of metabolic pathways and their integration with broader tumor biology present challenges that require further investigation. Future research should focus on elucidating these intricate mechanisms and exploring innovative strategies to modulate its metabolic pathways for clinical applications.

In our research, we identified elevated piperine levels as a risk factor for GC, even after controlling for other metabolite effects. Previous studies have demonstrated piperine’s cytotoxic effects on tumor cells across various cancer models [52–54], including its ability to impact apoptosis and proliferation in GC cells via the ROS-mitochondria-associated signaling pathway [55]. Piperine, an alkaloid found in black pepper (Piper nigrum), is widely studied for its pharmacological properties, including anti-inflammatory, antioxidant, and anti-cancer activities [56, 57]. However, its potential pro-tumorigenic effects, particularly in the context of GC, warrant further investigation. Mechanistically, piperine-induced ROS generation is associated with oxidative stress, which can lead to oxidative DNA damage and activate pro-inflammatory pathways such as NF-κB and STAT3, both implicated in inflammation-driven carcinogenesis and tumor progression [58, 59]. Additionally, piperine’s inhibitory effects on cytochrome P450 enzymes, particularly CYP1A1 and CYP2E1, may influence the metabolism of procarcinogens like benzo[a]pyrene, potentially increasing the formation of DNA adducts and heightening mutagenic risk and gastric mucosal damage [64, 66]. Piperine’s interaction with drug transport proteins, such as P-glycoprotein, may also alter the bioavailability of various compounds, including carcinogens, further influencing cancer risk [60, 61].

The discrepancy between our findings and earlier studies could stem from two key factors. First, previous experimental research has predominantly focused on GC cell lines, which may not reflect the prolonged impacts of piperine on normal gastric mucosal cells. Second, variations in study cohorts might have contributed to differing outcomes, as our study primarily included participants from Finland and the UK, emphasizing genetic and environmental differences compared to other populations. Despite these complexities, piperine shows potential as both a biomarker and a therapeutic agent in GC. Its modulation of oxidative stress and inflammatory pathways offers opportunities for early detection and the development of targeted interventions. Future research should prioritize understanding piperine’s biological mechanisms in both normal and cancerous cells. Advanced preclinical models, such as organoids and patient-derived xenografts, will be crucial to elucidate its dual effects. Furthermore, exploring piperine’s metabolic pathways and interactions with other biomolecules will be essential to assess its clinical relevance in GC prevention and treatment.

Additionally, we recognized Phe-Phe dipeptide as a potential protective agent against GC, even after controlling for other metabolite effects. Currently, no research has directly investigated the link between Phe-Phe dipeptide and GC. A limited number of studies have analyzed its correlation with other diseases, yielding inconsistent results. Yang et al. reported decreased levels of Phe-Phe dipeptide in thyroid cancer patients [62]. In contrast, Chen et al. associated increased levels with a heightened risk of lung cancer and a reduced risk of pulmonary tuberculosis [63]. Furthermore, a comprehensive epidemiological study by Stolzenberg-Solomon et al. found that higher concentrations correlated with a greater incidence of pancreatic cancer [60], reflecting its complex role in disease progression.

Phe-Phe dipeptide, composed of two phenylalanine residues, is a metabolic intermediate formed during protein degradation. Dipeptides are known to exert various biological activities, including antioxidant properties and modulation of cellular signaling pathways, which may influence tumorigenesis [61]. Phe-Phe dipeptide, composed of two phenylalanine residues, is a metabolic intermediate formed during protein degradation. Recent advances suggest that short peptides, including Phe-Phe, can influence mitochondrial functions and trigger apoptotic signaling under specific conditions. Studies have shown that peptides derived from protein degradation may self-assemble within mitochondria, disrupting mitochondrial membrane potential and inducing selective apoptosis in cancer cells while sparing normal cells [64]. This mechanism underscores the potential for short peptides to regulate cellular energetics and redox balance, which could help inhibit tumor growth.

The inconsistent findings regarding Phe-Phe dipeptide's role in different diseases may arise from two primary factors. First, its biological effects may vary depending on tumor type and tissue-specific metabolism. Second, challenges in controlling confounders and addressing reverse causation in conventional observational studies could contribute to these discrepancies. Our findings indicate that elevated levels of Phe-Phe dipeptide are associated with a reduced risk of GC, suggesting its potential as a therapeutic target for modulating metabolic pathways involved in GC pathogenesis. By influencing key metabolic processes, this dipeptide may contribute to strategies aimed at preventing GC development or improving treatment outcomes. Future research should focus on addressing these knowledge gaps through comprehensive investigations into the biological and clinical significance of Phe-Phe dipeptide. Multi-omics approaches, such as metabolomics and proteomics, could elucidate its interactions with tumor metabolism and signaling pathways. Additionally, large-scale epidemiological studies and randomized controlled trials will be crucial to validate its role and explore its application in GC prevention and treatment.

Our MR study provides several important strengths. First, it examines a broad range of human blood metabolites (initially 486, with 485 retained), making it one of the most comprehensive investigations to date into the metabolic factors that may influence GC risk. By using genetic variants as instrumental variables, our MR approach minimizes confounding and reverse causation, offering more reliable causal inferences than traditional observational studies. We applied stringent SNP selection criteria and performed multiple sensitivity analyses, including tests for pleiotropy, heterogeneity, and the correct causal direction, to ensure the robustness of our findings. The absence of horizontal pleiotropy or significant heterogeneity, along with the consistency of results confirmed by replication in independent GWAS data, further bolsters our confidence in the conclusions. Additionally, LDSC regression supported our findings by examining genetic correlations and heritability. Overall, these measures strengthen the credibility of our MR-based insights, providing a solid foundation for identifying potential metabolic biomarkers and therapeutic targets in GC.

However, some limitations persist. Firstly, the limited count of relevant SNPs identifiable at the genome-wide scale necessitated the use of a moderately relaxed MR analysis cutoff, a tactic frequently adopted in comparable studies. Yet, the F-statistic for all chosen SNPs surpassed 10, indicating robust IVs. The direction of causality, validated by the reverse MR Steiger test, supports this approach. Secondly, due to the original data's categorization, we couldn't delve deeper into GC's specific pathological or molecular subtypes, limiting us to a broader GC analysis. Thirdly, our MR study predominantly utilized GWAS data from European descendants, which limits the generalizability of our findings to other ethnic groups, as genetic architecture and environmental exposures vary significantly across populations. Future studies should include more diverse datasets to validate these results and explore population-specific differences. Additionally, excluding metabolites with insufficient SNP instruments may have narrowed the scope of discoveries. Integrating genomic data with transcriptomic or metabolomic datasets could help identify additional genetic variants, enabling a more comprehensive analysis of metabolite-GC relationships. Fourthly, MR estimate accuracy hinges on sample size, indicating that a larger sample could refine our causal insights between metabolites and GC. Fifthly, despite adjusting for statistically significant interactions among metabolites through multivariate analysis, the complexity of the human metabolic network and pathways remains a challenge. Metabolites that did not reach statistical significance could still influence the three metabolites under study. Furthermore, the significance of interactions among metabolic products cannot be underestimated. The influence of individual metabolic products on gastric cancer could potentially introduce biases into our analysis. Sixthly, of the 485 metabolites studied, instead of adjusting the MR estimates for multiple testing, we leveraged two GWAS datasets for replication and meta-analysis to ensure the results' robustness. The strict multiple-testing threshold may overlook connections notable in solitary studies, so we included potential metabolites with *p* < 0.05 linked to GC. Seventhly, our reliance on summary-level GWAS data rather than individual-level data limits the ability to adjust for potential confounders and may introduce bias due to unaccounted population stratification. Finally, the biological mechanisms by which the identified metabolites influence GC risk remain incompletely understood, limiting definitive causal conclusions. Beyond the complexity of pinpointing causal relationships, translating these genetic and metabolomic insights into clinical interventions faces multiple challenges. These include ensuring metabolite stability, understanding pharmacokinetics within intricate metabolic networks, and accounting for dynamic regulatory processes that integrate multiple pathways. Additionally, effective therapeutic modulation of metabolic reprogramming may require coordinated targeting of several metabolic nodes rather than a single metabolite. Future research should focus on multi-omics integration, advanced metabolic modeling, and validation of biomarkers in controlled trials, paving the way for personalized therapeutic strategies. While Mendelian randomization provides valuable etiological insights, rigorous fundamental research and carefully designed clinical studies are vital to fully harness these findings and guide their incorporation into routine clinical practice.

## Conclusions

In conclusion, our MR study elucidated causal relationships between three metabolites(3-methyl-2-oxovalerate, piperine, Phe-Phe dipeptide) and GC. These findings offer critical insights for GC's early screening, prevention, and treatment, and inform the direction of subsequent research efforts.

## Supplementary Information

Supplementary Material 1.

Supplementary Material 2.

## Data Availability

Data is provided within the manuscript and supplementary information files.

## Abbreviations

GC: Gastric cancer
ROS: Reactive oxygen species
GWAS: Genome-wide association study
SNPs: Single nucleotide polymorphisms
MR: Mendelian randomization
RCTs: Randomized controlled trials
IVs: Instrumental variable
IVW: Inverse Variance Weighted
WM: Weighted Median
MR-PRESSO: MR-Pleiotropy RESidual Sum and Outlier
OR: Odds ratio
LDSC: Linkage Disequilibrium Score
MVMR: Multivariable Mendelian randomization
BCKA: Branched-chain ketoacid
BCAA: Branched-chain amino acid
