# Supplementary material for: Human blood metabolites and gastric cancer: a Mendelian randomization analysis
Source: BMC Gastroenterol. 2024 Dec 30;24:478. doi: 10.1186/s12876-024-03576-2 (PMC11684236; doi:10.1186/s12876-024-03576-2)
Supplement: Supplementary file 1 — Supplementary Material 1. [file 12876_2024_3576_MOESM1_ESM.docx]

***Additional Material***

**Supplementary Figures**

**Supplementary Figures, Additional file 1: Forest plots for the Mendelian randomization leave-one-out analysis of the significant inverse variance weighted estimates.**

**
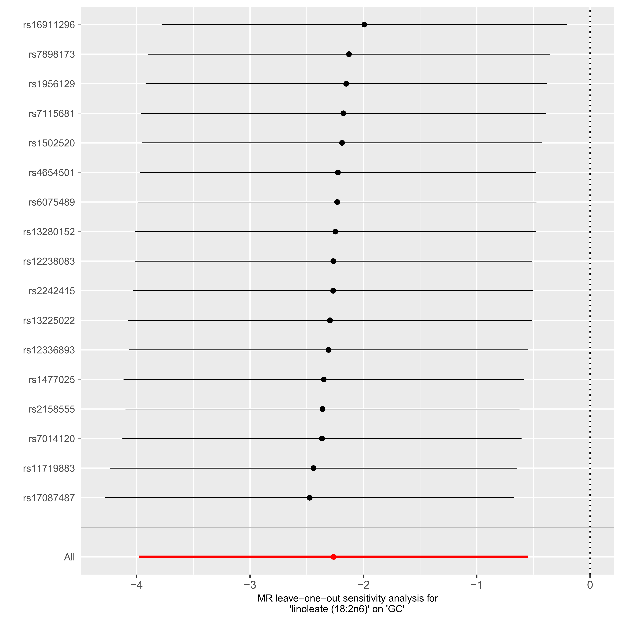

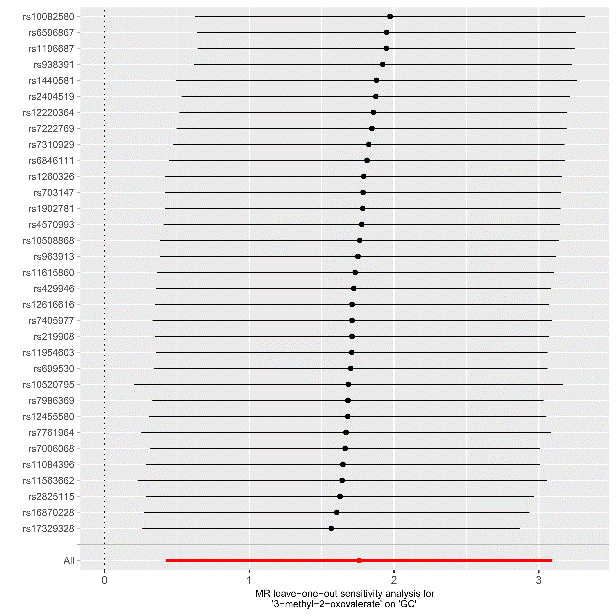
**

**
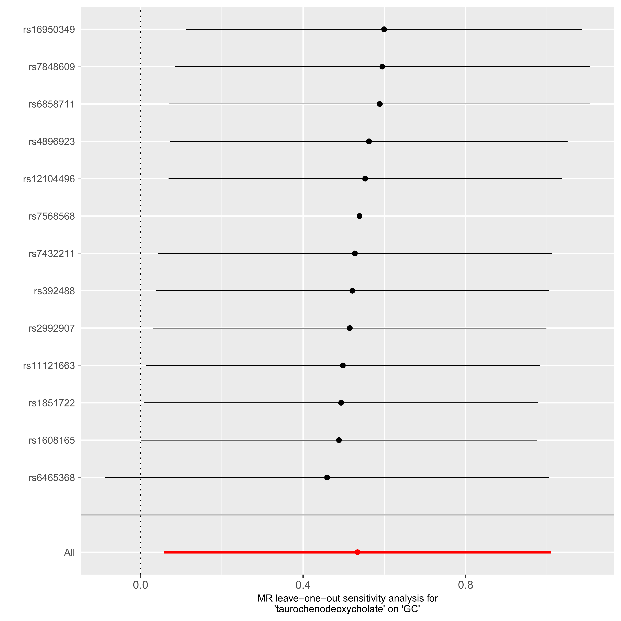

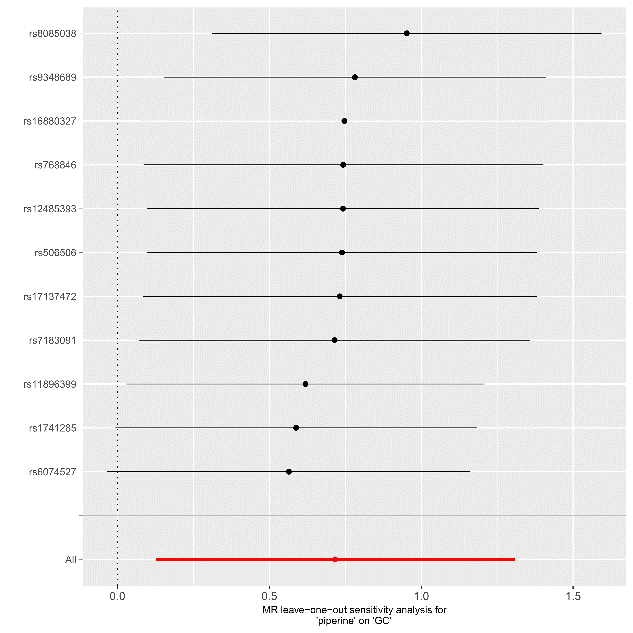
**

**
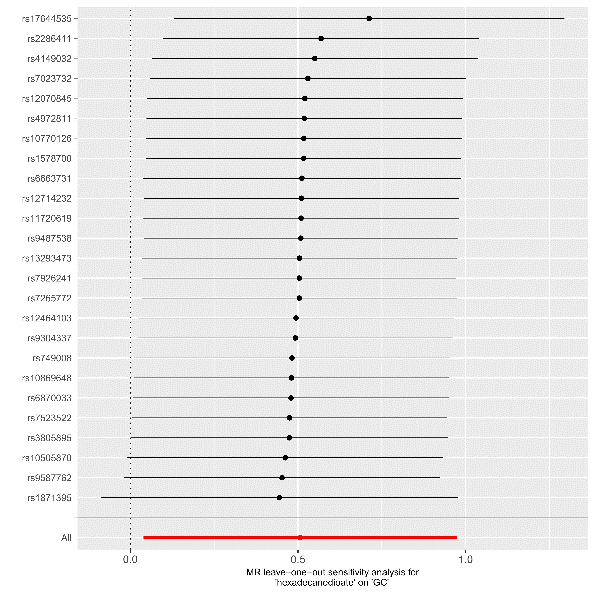

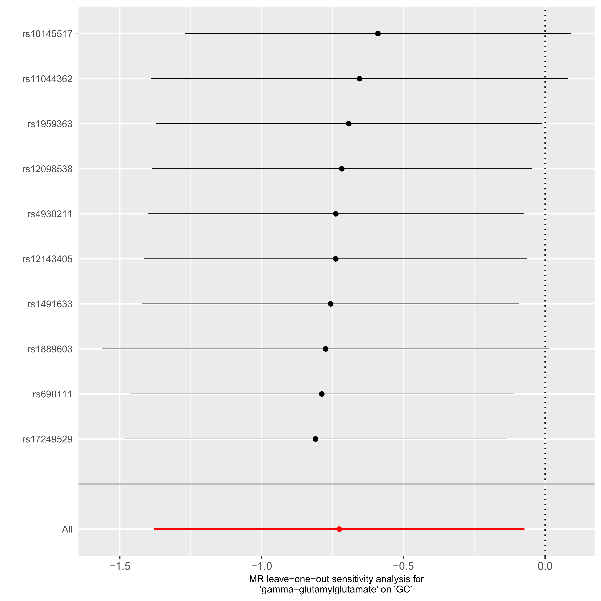
**

**
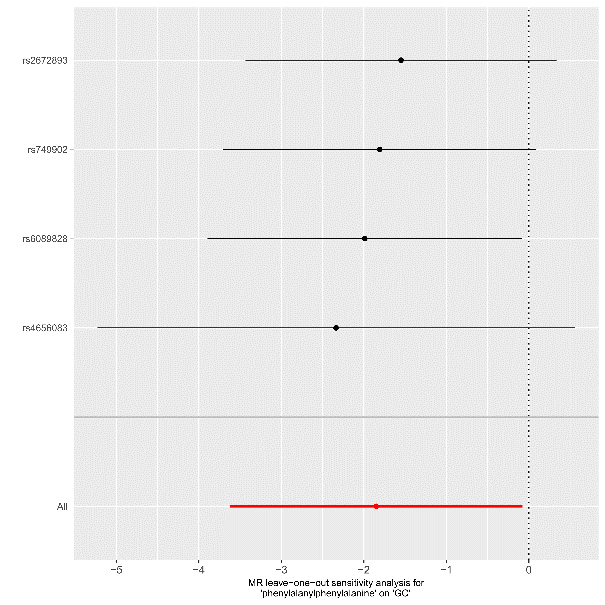
**
